# Supplementary figures and images for: High-throughput, single-copy sequencing reveals SARS-CoV-2 spike variants coincident with mounting humoral immunity during acute COVID-19
Source: PLoS Pathog. 2021 Apr 8;17(4):e1009431. doi: 10.1371/journal.ppat.1009431 (PMC8031304; doi:10.1371/journal.ppat.1009431)

**A**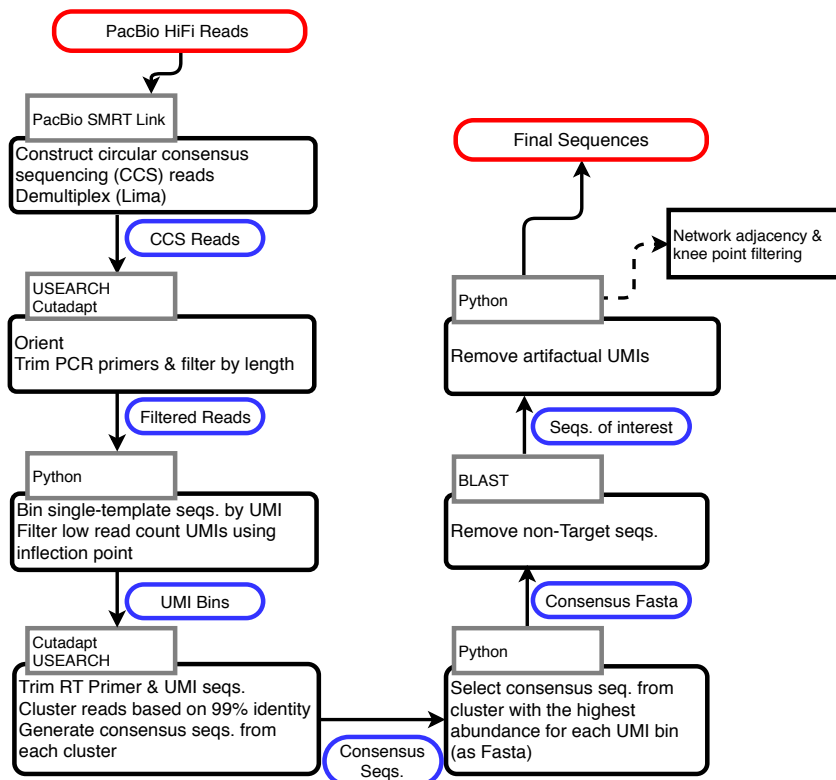**B**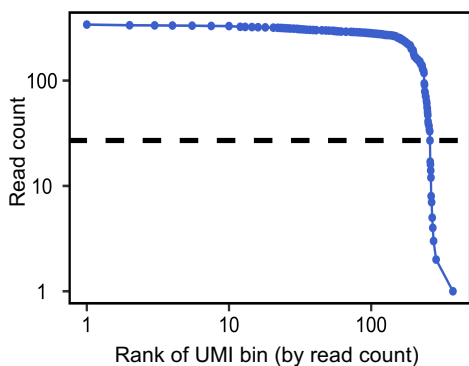**C**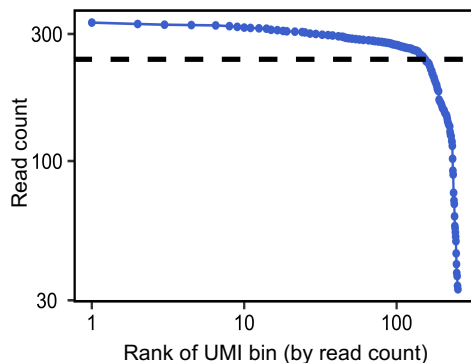

Supplement: S2 Fig — (A) Bioinformatic pipeline, depicting sequential workflow steps and tools used. Black boxes show tasks at each step, with the tools used in the grey boxes, and the outputs in the blue bubbles. (B) Initial exclusion of false UMI bins based on read count distribution on a log scale. The dashed line indicates the read count inflection point below which UMI bins in this sample were excluded. (C) Final exclusion of low count UMI bins based on read count distribution on a log scale. The dashed line indicates the read count knee point below which UMI bins in this sample were excluded, following initial false bin removal from the sample and network adjacency. Data are presented for the cultured virus sample presented in Fig 2. (PDF) [file ppat.1009431.s002.pdf]

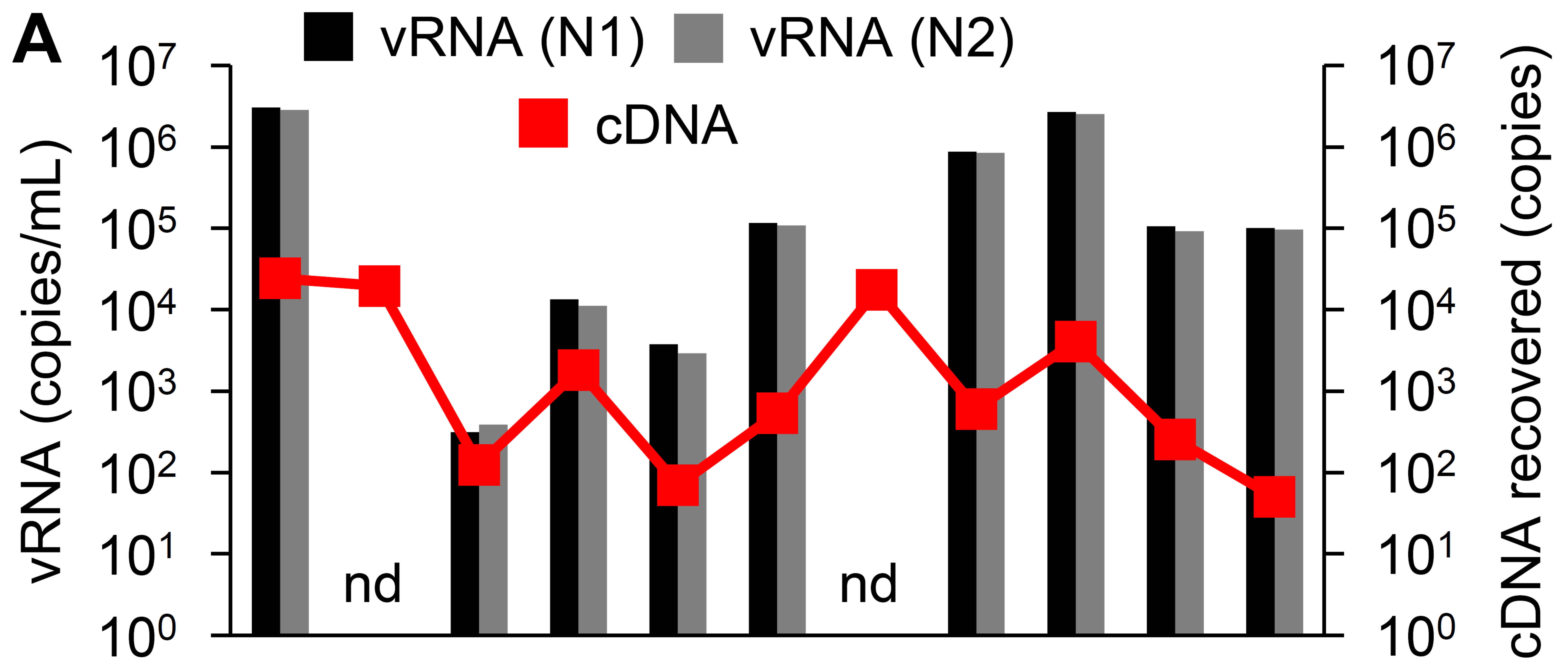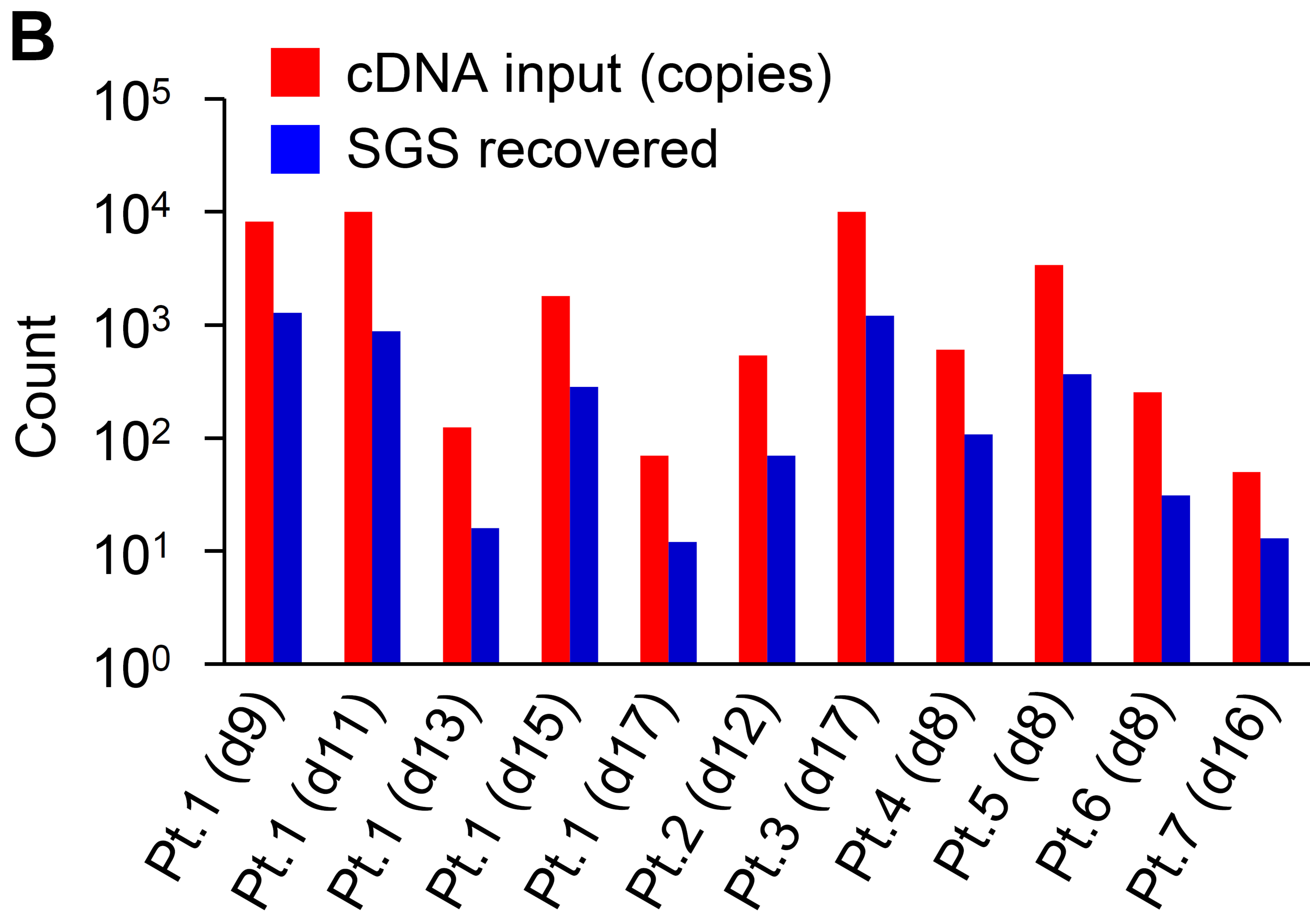

Supplement: S3 Fig — (A) Comparison of virus load of original sample with total cDNA synthesis yield. (B) Comparison of cDNA input copies from each sample with final SGS counts. (PDF) [file ppat.1009431.s003.pdf]

**A**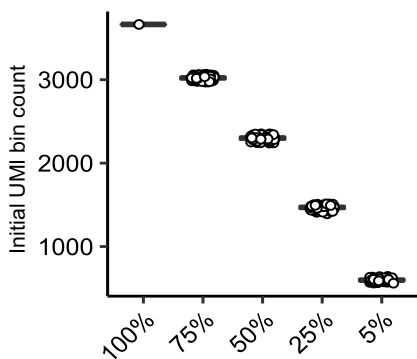**B**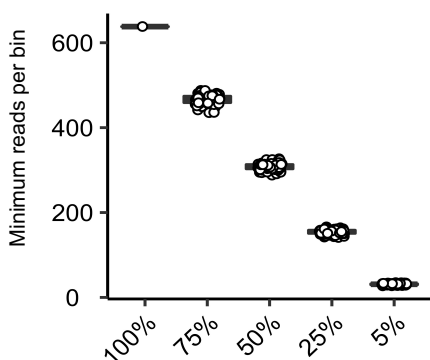**C**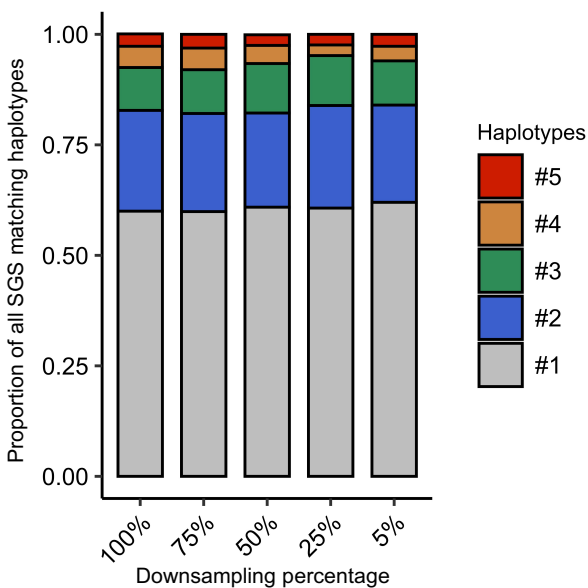

Supplement: S4 Fig — Each subsample was generated by random draws of a fixed percentage from reads without replacement. This process was repeated 100 times for each percentage. (A) The initial numbers of UMI bins (y-axis) are shown for different degrees of downsampling (x-axis). (B) The minimum read counts per UMI bin (y-axis) are shown for different degrees of downsampling (x-axis). (C) Proportion of each haplotype present in the 100% sample and in each subsample. Data analyzed are from sequencing of participant 1, day 15. (PDF) [file ppat.1009431.s004.pdf]
