# Supplementary material for: High-throughput, single-copy sequencing reveals SARS-CoV-2 spike variants coincident with mounting humoral immunity during acute COVID-19
Source: PLoS Pathog. 2021 Apr 8;17(4):e1009431. doi: 10.1371/journal.ppat.1009431 (PMC8031304; doi:10.1371/journal.ppat.1009431)
Supplement: S1 Fig — SARS-CoV-2 genomic RNA (gRNA) is reverse-transcribed with a primer that binds in ORF6, downstream of the M gene stop codon, and includes a UMI sequence of 8 random nucleotides flanked by a PCR reverse primer binding site. Reverse-transcription products are amplified by PCR using a forward primer that binds in ORF1, upstream of the spike gene start codon. Amplified products are then subjected to long-read sequencing. (PDF) [file ppat.1009431.s001.pdf]

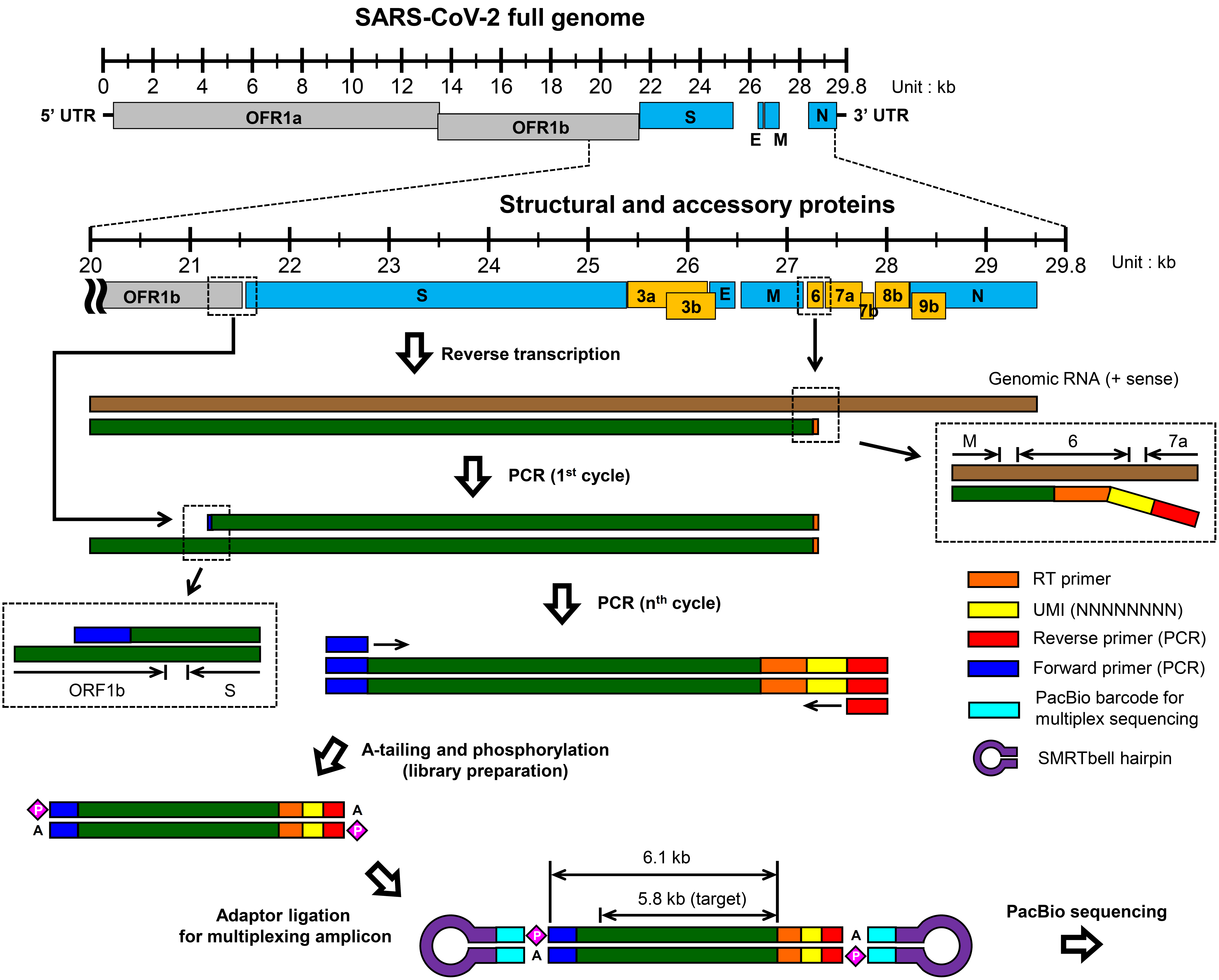

SARS-CoV-2 full genome
0 2 4 6 8 10 12 14 16 18 20 22 24 26 28 29.8
Unit : kb
5' UTR
OFR1a
OFR1b
S
E
M
N
3' UTR
Structural and accessory proteins
20 21 22 23 24 25 26 27 28 29 29.8
Unit : kb
OFR1b
S
3a
3b
E
M
6
7a
7b
8b
9b
N
Reverse transcription
Genomic RNA (+ sense)
M
6
7a
PCR (1st cycle)
ORF1b
S
PCR (nth cycle)
RT primer
UMI (NNNNNNNN)
Reverse primer (PCR)
Forward primer (PCR)
PacBio barcode for multiplex sequencing
SMRTbell hairpin
A-tailing and phosphorylation (library preparation)
P
A
Adaptor ligation for multiplexing amplicon
6.1 kb
5.8 kb (target)
PacBio sequencing
