# Supplementary material for: High-throughput, single-copy sequencing reveals SARS-CoV-2 spike variants coincident with mounting humoral immunity during acute COVID-19
Source: PLoS Pathog. 2021 Apr 8;17(4):e1009431. doi: 10.1371/journal.ppat.1009431 (PMC8031304; doi:10.1371/journal.ppat.1009431)
Supplement: S1 Table — (PDF) [file ppat.1009431.s005.pdf]

Pt.	*Age*	*Sex*	*Race/Eth.*	*Sample Day**	*Comorbidity*	*Suppl. O_2*	*Pharmacotherapy*	*Outcome*
1	38	F	H	9, 11, 13, 15, 17	LCL, HSCT	None	None	Recovered
2	60	M	AA	12	Chordoma	None	None	Recovered
3	75	M	H	17	Dyslipidemia	MV	Tocilizumab Glucocorticoids	Died
4	54	M	W	8	Asthma, CLL	MV	Remdesivir	Recovered
5	67	M	AA	8	Obesity	MV	Glucocorticoids Remdesivir	Died
6	22	M	W	8	None	HFNC	None	Recovered
7	62	M	Asian	16	GERD, OSA	MV	None	Recovered

*Sample Day denotes day of clinical illness.

H, Hispanic; AA, African-American; W, white; LCL, large cell lymphoma; HSCT, hematopoietic stem cell transplant; CLL, chronic lymphocytic leukemia; GERD, gastro-esophageal reflux disease; OSA, obstructive sleep apnea; MV, mechanical ventilation; HFNC, high-flow nasal cannula.
