# Supplementary material for: High-throughput, single-copy sequencing reveals SARS-CoV-2 spike variants coincident with mounting humoral immunity during acute COVID-19
Source: PLoS Pathog. 2021 Apr 8;17(4):e1009431. doi: 10.1371/journal.ppat.1009431 (PMC8031304; doi:10.1371/journal.ppat.1009431)
Supplement: Attachment — Submitted filename: Ko_ResponseLetter_02222021corrected.docx [file ppat.1009431.s007.docx]

Revision list for # PPATHOGENS-D-21-00031

**High-Throughput, Single-Copy Sequencing Reveals SARS-CoV-2 Spike Variants Coincident with Mounting Humoral Immunity during Acute COVID-19**

Sung Hee Ko^¶^, Elham Bayat Mokhtari^¶^, Prakriti Mudvari^¶^, Sydney Stein,

Christopher D. Stringham, Danielle Wagner, Sabrina Ramelli, Marcos J. Ramos-­Benitez,

Jeffrey R. Strich, Richard T. Davey, Jr., Tongqing Zhou, John Misasi, Peter D. Kwong,

Daniel S. Chertow, Nancy J. Sullivan, and Eli A. Boritz^*^

^¶^These authors contributed equally to this work.

^*^[boritze@mail.nih.gov](mailto:boritze@mail.nih.gov) (EAB)

**Reviewer #1:**

***Summary:*** *Ko et al. develop a new method for sequencing single virus genomes with long-read technology and apply it to a cell culture adapted virus and to 7 clinical SARS-CoV2 samples. The sequencing method developed in this manuscript seems quite useful, with the clear benefit of enabling haplotype resolution and mutation linkage. The authors also present an interesting case of within-host evolution that is correlated to antibody development. This is of particular interest now and provides a useful contribution to the discussion of the role of immune-compromised hosts to SARS-CoV2 evolution. Overall, I think that the authors conclusions and findings are reasonable and interesting, and that the method could be quite useful. However, some of the results are currently difficult to interpret without some additional controls to validate the sequencing method for its robustness across viral RNA input copies and total number of output single consensus genomes. I suggest some specific controls that I think would be useful for clarifying these points, as well as some points in the Methods that could be further clarified.*

**Author reply:** We appreciate these positive comments. The new manuscript version now contains experimental data in Table I, Table II, S3 Fig, and S4 Fig, as well as Results sections on “Validation of HT-SGS for SARS-CoV-2 Surface Protein Gene Sequencing” and “HT-SGS Performance in Direct Ex Vivo Sequencing of SARS-CoV-2.” This new material addresses specific questions about interpretation and methodological validation, and is discussed in detail below.

***Major comments***
***Comment 1.*** *The authors document very reasonable and extensive bioinformatic controls for sequence quality, UMI consolidation, and consensus genome calling. However, the authors have not directly performed validation experiments that benchmark a. this method’s intrinsic error rate on a sample with no variation or b. its ability to correctly characterize within-host haplotype frequencies across various RNA input quantities and read counts. In my opinion, the authors need to perform some additional validation experiments to address these concerns. To address point a, the authors should perform a simple validation experiment in which the authors sequence a clonal RNA transcript and report the number of detected haplotypes and their frequencies. This would provide a raw error rate of their method, and provide a more direct way of measuring the error rate of their RT and PCR protocols than by relying on the variant calling and error correction method described in the Methods. Suggestions for addressing point b are below in comments 2 and 3.*

**Author reply:** We agree with these points. In response to point a, we applied the HT-SGS process to a mixture of two clonal *in vitro* transcription products, one generated from a plasmid bearing the GenBank MN985325 (WA-1) sequence of our target region, and the other from a “double-mutant” version in which the sequences of two 20-base sections at the ends of the target region were scrambled (described in Materials and Methods). Without performing variant calling or excluding UMI bin consensus sequences that were unique within the dataset, we measured an error rate of 1.49 per 6115 bases (0.024%) for the WA-1 target and 1.56 per 6115 bases (0.025%) for the double-mutant target. Importantly, these putative errors included both single-nucleotide substitutions and insertions/deletions (indels), and likely represented a combination of true error (reverse-transcription error, PCR amplification error, mutations introduced during amplicon library preparation, and sequencing error) and other factors (*in vitro* transcription error, plasmid mutations). Moreover, when variant calling and exclusion of unique sequences were applied, we found that all resulting sequences matched their corresponding reference with no errors. We have now added these data and arguments to the Results (section on “Validation of HT-SGS for SARS-CoV-2 Surface Protein Gene Sequencing,” lines 109-119, as well as Table I and Fig 1B).

***Comment 2.*** *Given the known sensitivity of within-host variant detection to input RNA copies, the authors should report in a table somewhere in the main text the number of RNA copies in each sample that is sequenced.*

**Author reply:** We agree with this suggestion, and have now included Table II showing virus load, total cDNA copies recovered, input cDNA used for sequencing, and final SGS yield from all participant samples used in the study, with graphical representation of relationships among these data now shown in S3 Fig. Reasons for the discrepancy between RNA copies (detected by short amplicons in the N gene) and cDNA copies or SGS recovered are now discussed in Results (section on “HT-SGS Performance in Direct Ex Vivo Sequencing of SARS-CoV-2,” lines 149-161).

***Comment 3.*** *In the authors sequencing results, there is a wide range of the number of total single-genome consensus sequences generated for each sample. While in some samples the number is reasonably high >1000), in patients 6 and 7 and patient 1 on days 13 and 17, the total number of single genomes is quite low. The sequencing results from these samples are quite difficult to interpret as is. It seems to me that greater single genome consensus sequences should provide higher resolution of within-host haplotype frequencies, and I would guess that samples with higher viral RNA contents generally produce more single genome consensus sequences. However, these points are not made explicit and the authors have not designated a cutoff for either a viral load necessary for this method to be successful or for a number of successfully sequenced single genome consensus sequences for this method to accurately represent the within-host diversity of the sample. These values will be quite important for readers who may be interested in applying this new method, and are also important for interpreting the results shown in the paper. I would suggest that following revisions and control experiments, or something that accomplishes similar goals:*

*a. The authors should discuss in the text whether the number of single genome consensus sequences is correlated to the viral load of the original sample. A plot showing this relationship would be helpful. This is particularly important because the authors remark throughout the paper that their method can be used to characterize thousands of individual viral genomes, but they only ever report, at maximum, ~1200 single genomes for a given sample.*

**Author reply:** We appreciate this suggestion, and have now addressed the relationship between sequence yield and RNA input template number in Table II and S3 Fig. As shown in these display items, the number of single genome consensus sequences recovered by our method was correlated to the viral load of the original sample. Reasons for the discrepancy between RNA copies (detected by short amplicons in the N gene) and cDNA copies or SGS recovered are now discussed in Results (section on “HT-SGS Performance in Direct Ex Vivo Sequencing of SARS-CoV-2,” lines 149-161). To avoid confusion, we have also removed wording about “thousands of genomes” from the manuscript. Although we feel comfortable that many thousands of genomes can be sequenced accurately using our approach, limited largely by quantity of sample and sequencing capacity/cost, we acknowledge that the manuscript does not address this and appreciate the reviewer’s calling attention to this.

*b. The authors could perform some sort of serial dilution experiment in which a known variant haplotype is spiked into a wild type, clonal RNA at some frequency, and then serial dilutions are generated and sequenced. This would provide important information about how to interpret haplotype frequencies derived from low copy number samples. I realize that this is bit more work, but it would provide information that would be quite useful. Alternatively, if the authors can perform some sort of experiment that clarifies the relationship between viral load and Method accuracy, that would be great.*

**Author reply:** We agree with this suggestion, and have now included results from the suggested experiment in Table I, with description in Results (section on “Validation of HT-SGS for SARS-CoV-2 Surface Protein Gene Sequencing,” lines 109-119). Although yields of the two different sequences were not perfectly balanced at a 1:1 input ratio (likely due to pipetting error), two serial 10-fold dilutions in either direction demonstrated appropriate dose-responses. This experiment was also used to evaluate inter-template recombination, with results of that evaluation included in Table I and discussed in the same Results section.

*c. The authors should assess in some way how the total number of unique single genome consensus sequences impacts haplotype detection. This could be accomplished bioinformatically by randomly subsampling down one of their samples with >1000 single genome consensus sequences to varying degrees, and comparing the output. This sort of information would be really helpful for interpreting the haplotype results for the samples with very low numbers of single genome consensus sequences.*

**Author reply:** We thank the reviewer for this helpful comment. To analyze how the total number of unique single genome consensus sequences impacts haplotype detection, we subsampled at varying percentages (5%, 25%, 50%, 75%) from the participant 1, day 15, cell pellet fraction raw sequencing data. To achieve this, we randomly drew a fixed percentage from the reads without replacement, repeating 100 times per percentage. Then, we processed each subsample through our bioinformatic pipeline and examined the effect of downsampling on the initial UMI bin count and the knee point cutoff (minimum reads per bin) across downsampling percentages. As expected, downsampling caused a dose-dependent reduction in both the initial (unprocessed) UMI bin counts and minimum reads per bin (S4A and S4B Fig). Next, for each downsampling percentage, we selected the downsample with final UMI bins closest to the median number of final UMI bins. This was done to ensure that the chosen downsamples were reasonable representatives of the downsampling group. Subjecting these four selected downsamples to variant calling and haplotype detection, we observed that all the haplotypes detected in the whole sample were also detected by the downsamples, in very similar proportions (S4C Fig). This shows that the combination of filtering criteria in our pipeline (inflection, network, and knee point) resulted in UMI bins that included haplotypes independent of downsampling percentage across a wide range. These results are described in lines 163-167 of the revised manuscript.

***Minor comments***
***Comment 1.*** *In the Methods, the authors report using 1000 cDNA input copies into the PCR reaction. This point was not clear to me. The authors are able to generate >1000 unique single genome consensus sequences for some samples, despite only using 1000 cDNA input copies. How is this possible? Is this just pipetting error? Why did the authors choose to limit their input to 1000 cDNA copies? The authors state multiple times in the manuscript that their method is designed to sequence thousands of individual viral genomes, but it seems that by limiting the copy numbers that they are by definition restricting their sequencing to ~1000 copies. The authors should clarify this in the text.*

**Author reply:** We thank the reviewer for pointing this out. The statement about 1000 cDNA input copies/reaction in the Materials and Methods was an error, and has now been removed. Table II in the revised manuscript now shows the input cDNA copy number for sequencing of each sample, as described in response to comment 3a.

***Comment 2.*** *If possible, I would find it very helpful to see a calculation of: given the number of input RNA molecules, what is the probability that 2 different RNAs get labelled with the same UMI? Alternatively, how much in excess is the UMI vs the RNA? Some sense for this would be helpful.*

**Author reply:** We thank the reviewer for this comment. We investigated the possible UMI collision (two distinct molecules labeled with the same UMI) based on the assumption of uniformly distributed UMIs. Following Fu et al. (ref. 43), the expected number of unique UMIs captured is k =$m[1-e^{-\frac{n}{m}}]$, where n is the number of molecules and m is the size of UMI pool. Therefore, $n=-m \ln(1-\frac{k}{m}).$ In our study we used 8-base UMIs, hence, we have UMI pool of size $4^{8}\approx65000$ to sample from. Given the number of observed unique UMIs in a particular sample, we can estimate the number of molecules, therefore we expect to observe (n-k) collisions. This number was calculated in each of the samples and observed to be small. The probability of collision was calculated to be at most 4%, with an average 1.8% across all samples. We also note that, in the event of a UMI collision between two distinct sequences, our clustering and consensus formation process results in preservation of the sequence cluster with higher read abundance and removal of the sequence cluster with lower read abundance. This description has been added to the Materials and Methods (section on “UMI Collision Estimates,” lines 419-430).

***Comment 3.*** *On lines 211-216, the authors discuss the amount of virus in patient 1 as comparable to other, acutely infected patients, but do not discuss the duration of shedding. While the viral load is certainly important for within-host viral evolution, the duration of shedding is also critical given that it determines whether within-host viral replication is still occurring at the time of antibody development. The authors should acknowledge this and perhaps could cite some literature describing within-host evolution during prolonged infections.*

**Author reply:** We thank the reviewer for this comment. The revised manuscript now addresses this issue in the Introduction (lines 56-61) and Results (219-221), and cites several studies showing virus evolution in cases of prolonged infection, as well as a larger analysis of mutations in recurrently deleted regions (RDRs) that was published after our initial submission.

***Comment 4.*** *The description of the node collapsing and adjacency method could be clarified a bit. It sounds to me like what is happening is that you connect UMIs that are separated by 1 and meet this number criteria and then you collapse them all into 1. Is that right? It is also not immediately clear to me what the difference between the knee point and inflection point is. The authors could add a sentence in the Methods to describe this for readers who may not be familiar with this.*

**Author reply:** Yes, the reviewer is correct, and we appreciate this comment. We executed this algorithm iteratively, however we did not collapse the UMIs, but instead removed presumptive false UMIs. The knee point is the point of maximum curvature and inflection point is where the derivative is minimized, both serving as points distinguishing two groups of the count distribution. This is now depicted visually in S2 Fig. Inflection point is used initially as a way of removing bins with very low counts that we consider highly likely to represent error. Subsequently, after application of the network adjacency method for removal of false UMIs, the knee point cutoff is applied to add an additional layer of stringency. The rationale for this high level of stringency is to ensure that interesting variant sequences documented in this study are distinguished from lower-count sequences. Descriptions of these details were added to the manuscript.

***Comment 5.*** *It is not clear to me exactly when the variant calling correction is implemented. Is this done on the consensus sequences of each UMI bin? Or is this done on the raw reads themselves?*

**Author reply:** We thank the reviewer for calling attention to this. Variant calling was done on the consensus sequences of the UMI bins, with the goal of filtering out reverse transcription errors and other rare errors that would theoretically not be identified in the UMI-based consensus formation process, and that could lead to the reporting of false sequences in the manuscript if not addressed. We have now modified Fig 1A to represent more clearly all steps in the analysis process, and hope that the modifications are helpful.

***Comment 6****. On line 263: “Supernatants were transferred to separate tubes, and supernatant and pellet fractions were processed in parallel, although single-genome sequences derived from these two fractions were subsequently found to be similar and were thus pooled for each sample in the final analysis.” Do the authors mean that the pellets and supernatants were combined for one particular set of samples, or that the reads were combined later? Were pellets and supernatants initially separated to compare within-host variants in genomic RNA vs. cell-associated RNA?*

**Author reply:** We performed sample preparation for supernatant and pellet fractions separately using identical protocols, and then sequenced resulting libraries separately using different PacBio multiplexing barcodes. The goal of separating supernatant and pellet was to ensure that potential low-diversity sequence populations arising from a small number of infected cells with high vRNA copy number would not obscure greater levels of diversity present in free virions in the sample. However, upon examination of the sequence data, we found that supernatant and pellet datasets were very similar, with the same haplotypes represented in both. Therefore, for simplicity, supernatant and pellet sequences were combined in downstream analysis and data presentation. This is described in the revised Materials and Methods (lines 343-346).

***Comment 7****. On line 304, what is the “log rank of that UMI bin within the sample”? I had assumed initially that the log rank was just an ordered list of UMIs, ordered by read count, but was unsure. Also, is Figure S2 showing the results for the WA-1 BEI sample or some other sample?*

**Author reply:** The reviewer is correct, and this axis label has now been adjusted for clarity. The read count in each UMI bin was plotted against the rank of that UMI bin (on log scale) within the sample, and the inflection point (i.e., point of concavity change; revised S2B Fig) or knee point (revised S2C Fig) was calculated. As the reviewer suggests, results are shown for the WA-1 BEI sample. This has now been indicated in the revised legend.

**Reviewer #2:**

***Summary:*** *Ko et al. provide a timely report about sequence diversity in the Spike gene of SARS-CoV-2. They analyze both virus that has been grown in tissue culture and virus isolated from the respiratory tract from infected people. There is a critical need to follow Spike protein evolution both within and between people as this could lead to new strains that could escape the anticipated protection that will be provided by the current vaccines. The authors improve on existing sequencing approaches by using an SGS approach but at higher per-specimen sampling than is typically done, and this is done over a relatively large region of the genome (wedding the deep part of "deep sequencing" with SGS accuracy for sampling individual genomes). What has seemed like an obvious approach of using a UMI with PacBio long read technology has been difficult due to the high error rate of PacBio which makes it hard to accurately identify the UMI. Overcoming this problem will be a welcome addition to the field. The number of long reads one can get from a sample is limited by the number of long cDNA molecules that RT can generate (i.e. the number of templates), in this case 6.1 kb. The relatively high titers of SARS-CoV-2 samples conveniently overcomes this limitation by providing a larger number of starting RNA templates. The paper does an elegant job of linking the antibody response to longitudinal changes in the Spike protein sequence and antibody escape in one case but a case that is very informative. This is an important piece of work. I have only a few technical questions. The biology in the paper is on mark addressing important questions.*

***Minor comments***
***Comment 1****. The genomes appear to contain mostly single nucleotide polymorphisms with but maybe some examples of polymorphisms in two different genes. To get at the issue of recombination during PCR (which always happens) it would be good to be clear whether or not the linkage is perfect in the sequenced genomes or whether there has been any scrambling of the markers between genomes. Scrambling could have occurred in vivo or during PCR, but an absence of scrambling would indicate that it didn't happen during PCR. It still happens during PCR but the ability to build a consensus sequence for each template masks those events.*

**Author reply:** We appreciate this constructive suggestion. We have now applied the HT-SGS process described in the manuscript to a mixture of two clonal *in vitro* transcription products, one generated from a plasmid bearing the GenBank MN985325 (WA-1) sequence of our target region, and the other from a “double-mutant” version in which the sequences of two 20-base sections at the ends of the target region were scrambled and thus clearly distinguishable from the original. Without performing variant calling or excluding UMI bin consensus sequences that were unique within the dataset, we detected no recombinants in these experiments. Final numbers of SGS matching WA-1 and double-mutant references in these experiments (after variant calling and unique SGS exclusion, and obviously still including no recombinants) are now presented in revised Table I, with a description of the full experimental findings presented in the revised Results (“Validation of HT-SGS for SARS-CoV-2 Surface Protein Gene Sequencing,” lines 109-119, as well as Table I and Fig 1B). Overall, though these results do not rule out the potential for recombinant sequences in our approach – particularly recombinants that might arise during the reverse transcription step – they establish a maximum estimate of recombinant frequency for this target region at <1% of all recovered SGS.

***Comment 2.*** *Since cDNA synthesis has poor processivity it would be interesting to know how many genome sequences are obtained as a function of copies of RNA used in the initial cDNA reaction, i.e. what is the efficiency of template utilization in this sequencing approach?*

**Author reply:** This is an excellent point, particularly in the case of *ex vivo* respiratory samples where RNA quality and enzyme inhibitors can prove problematic. We have now addressed the issue of template utilization efficiency in Table II and S3 Fig. As shown in these display items, the number of single genome consensus sequences recovered by our method was correlated to the viral load of the original sample, but was considerably lower. Reasons for the discrepancy between RNA copies (detected by short amplicons in the N gene) and cDNA copies or SGS recovered are now discussed in Results (section on “HT-SGS Performance in Direct Ex Vivo Sequencing of SARS-CoV-2,” lines 149-161).

***Comment 3.*** *RT has a reported incorporation error rate of 1 in 10,000 nucleotides during cDNA synthesis (I believe listed in the manufacturers information). This would mean that every second genome would have an RT error incorporated. In this scenario a pool of identical RNA sequences would have the occasional difference due to RT during cDNA synthesis and still have its own UMI tag so artificial diversity. However, it seems that the authors are looking at homogeneous populations that defy this presumption. One way to address this question is to look at the same population twice. If the low level diversity is the result of RT error then it should not be reproducible. If it is real then it will repeat (given sufficient sampling). This is an important question both for the technique and for understanding true low level diversity in these viral populations.*

**Author reply:** We thank the reviewer for raising this extremely important point. To avoid presenting sequences containing unique reverse-transcription or other rare errors as real sequences in the manuscript, we used variant calling to flag uncommon mutations or those with specific error signatures (i.e., short insertions at homopolymer sites), and then reverted these to the sample consensus (described in revised manuscript Materials and Methods, lines 431-452). Furthermore, we removed individual SGS from our dataset that represented haplotypes occurring in only a single UMI bin (described in revised manuscript Materials and Methods, lines 415-418). To clarify these steps, we have now revised Fig 1A to present what was done more explicitly. Using this analytic approach, sequences derived from clonal RNAs produced by *in vitro* transcription of plasmid DNA showed no errors relative to the plasmid DNA reference sequences. We have added these data and arguments to the Results (section on “Validation of HT-SGS for SARS-CoV-2 Surface Protein Gene Sequencing,” lines 109-119), and to Table I.

Furthermore, we completely agree with the reviewer that accurate measurement of low-level diversity in these populations is very important, and would not want to give the impression that our findings rule out low-level diversity for samples where we only report a single haplotype. To address this, we have now added a new third paragraph to the revised manuscript discussion (lines 264-279, and quoted below). We feel that this additional discussion does a better job of explaining the limitations of approaches like ours to readers who are not highly experienced in sequence analysis.

*“It is important to emphasize that our bioinformatic strategy in this study prioritized the accuracy of reported variants over attempts to interpret very rare, potentially spurious sequences. Because the sensitivity of SGS depends in part on the number of genomes analyzed in each sample, future HT-SGS studies can achieve higher sensitivity for uncommon variants by preparing larger quantities of sample, by further optimizing virus RNA yield, integrity, and downstream processing efficiency, and by analyzing anatomic sites outside the upper respiratory tract that could harbor genetically distinct virus subpopulations (13, 27). However, accurate detection of rare variants in our HT-SGS process is also limited by the intrinsic error rate of the RT step. Based on a frequency estimate of approximately 0.00007 error/base (28), we anticipated that RT errors would introduce at least one incorrect base into every 2-3 sequences from our chosen target region in this study. Although the variant calling, rare mutation reversion, and unique SGS exclusion performed at the end of our analytical pipeline were sufficient to exclude such errors in validation studies while preserving key variants from in vitro and ex vivo virus populations, the use of these strategies precludes identification of real variants occurring at or below the level of RT error. Methodological refinements or new approaches that address this challenge in future studies will permit important insights into SARS-CoV-2 mutation rates in vivo.”*
